# Prevalence and Association of Trypanosomes and *Sodalis glossinidius* in Tsetse Flies from the Kafue National Park in Zambia

**DOI:** 10.3390/tropicalmed8020080

**Published:** 2023-01-21

**Authors:** Simegnew Adugna Kallu, Joseph Ndebe, Yongjin Qiu, Ryo Nakao, Martin C. Simuunza

**Affiliations:** 1 Department of Disease Control, School of Veterinary Medicine, University of Zambia, Lusaka P.O. Box 32379, Zambia; 2 College of Veterinary Medicine, Haramaya University, Dire Dawa P.O. Box 138, Ethiopia; 3 Department of Virology-I, National Institute of Infectious Diseases, Toyama 1-23-1, Shinjuku, Tokyo 162-8640, Japan; 4 Management Department of Biosafety, Laboratory Animal, and Pathogen Bank, National Institute of Infectious Diseases, Toyama 1-23-1, Shinjuku, Tokyo 162-8640, Japan; 5 Laboratory of Parasitology, Department of Disease Control, Faculty of Veterinary Medicine, Hokkaido University, N18 W9, Kitaku, Sapporo 060-0818, Japan; 6 Africa Centre of Excellence for Infectious Diseases of Humans and Animals, University of Zambia, Lusaka P.O. Box 32379, Zambia

**Keywords:** *Sodalis*, trypanosomes, tsetse flies, prevalence, association, Kafue National Park, Zambia

## Abstract

Tsetse flies are obligate hematophagous vectors of animal and human African trypanosomosis. They cyclically transmit pathogenic *Trypanosoma* species. The endosymbiont *Sodalis glossinidius* is suggested to play a role in facilitating the susceptibility of tsetse flies to trypanosome infections. Therefore, this study was aimed at determining the prevalence of *S. glossinidius* and trypanosomes circulating in tsetse flies and checking whether an association exists between trypanosomes and *Sodalis* infections in tsetse flies from Kafue National Park in Zambia. A total of 326 tsetse flies were sampled from the Chunga and Ngoma areas of the national park. After DNA extraction was conducted, the presence of *S. glossinidius* and trypanosome DNA was checked using PCR. The Chi-square test was carried out to determine whether there was an association between the presence of *S. glossinidius* and trypanosome infections. Out of the total tsetse flies collected, the prevalence of *S. glossinidius* and trypanosomes was 21.8% and 19.3%, respectively. The prevalence of *S. glossinidius* was 22.2% in *Glossina morsitans* and 19.6% in *Glossina pallidipes*. In relation to sampling sites, the prevalence of *S. glossinidius* was 26.0% in Chunga and 21.0% in Ngoma. DNA of trypanosomes was detected in 18.9% of *G. morsitans* and 21.4% of *G. pallidipes*. The prevalence of trypanosomes was 21.7% and 6.0% for Ngoma and Chunga, respectively. The prevalences of trypanosome species detected in this study were 6.4%, 4.6%, 4.0%, 3.7%, 3.1%, and 2.5% for *T. vivax*, *T. simiae*, *T. congolense*, *T. godfreyi*, *T. simiae* Tsavo, and *T. b. brucei*, respectively. Out of 63 trypanosome infected tsetse flies, 47.6% of the flies also carried *S. glossinidius,* and the remaining flies were devoid of *S. glossinidius*. A statistically significant association was found between *S. glossinidius* and trypanosomes (*p* < 0.001) infections in tsetse flies. Our findings indicated that presence of *S. glossinidius* increases the susceptibility of tsetse flies to trypanosome infections and *S. glossinidius* could be a potential candidate for symbiont-mediated vector control in these tsetse species.

## 1. Introduction

Tsetse flies (*Glossina*) are biological vectors of African trypanosomes which cause animal African trypanosomosis (AAT) and human African trypanosomosis (HAT) [1]. Tsetse flies occupy the ‘tsetse belt’ which covers an area of 10 million km^2^ that is about one third of the total land of the continent in 38 sub-Saharan African countries [2]. The density of the vector and the prevalence of trypanosome infections in the host is ascribed to complex interactions between and among humans, domestic livestock, wildlife, tsetse flies, trypanosomes and different economic and ecological factors [3].

AAT represents a group of vector-borne (*Glossina*) parasitic ailments in ruminants, camels, equines and carnivores which induce dramatic economic losses to animal producers as a result of mortality, morbidity and inefficient productivity [4]. Within the tsetse-infested areas, trypanosomosis reduces the offtake of meat and milk by a minimum of 50% [5]. The total loss of AAT to livestock productivity was estimated to be about USD 4.5 billion per year [6]. AAT is caused by several tsetse fly-transmitted trypanosome species including *Trypanosoma brucei brucei, Trypanosoma congolense, Trypanosoma simiae*, and *Trypanosoma vivax* [3,7,8].

Two trypanosome sub-species are responsible for HAT: *Trypanosoma brucei gambiense* and *Trypanosoma brucei rhodesiense* [9]. These cause high mortality in infected human population if left untreated. *Trypanosoma brucei gambiense* is found in 24 countries in west and central Africa [10]. This form of the disease currently accounts for over 98% of reported cases of sleeping sickness, transmitted through human-tsetse contact and causes a chronic infection. *Trypanosoma brucei rhodesiense* is found in 13 countries in eastern and southern Africa, representing less than two percent of reported cases [10]. This parasite has a complex transmission cycle involving a wide range of wildlife and livestock reservoirs and causes an acute infection in humans [10]. Even though 260 million people live in tsetse infested areas, only about 60 million are considered to be at risk of contracting the disease. The distribution of sleeping sickness areas has a focal nature and the localization of the actual areas fluctuates over the course of time [11,12].

Tsetse flies have established symbiotic relationship with maternally transmitted bacteria [13]. Many endosymbionts have been reported in various tissues of tsetse flies, but *Wigglesworthia glossinidia*, *Sodalis glossinidius* and *Wolbachia* species are the three major bacterial species that they harbor [14]. *Sodalis glossinidius* is found in the midgut, haemolymph, muscles, fat bodies, salivary glands, milk glands and reproductive system and so could interact with multiple species of trypanosomes that are harbored in different tissues [15]. *Sodalis* lacks a clearly defined functional role within its tsetse host [13]. However, it is suggested to play a role in facilitating susceptibility to trypanosome infection in tsetse by inhibiting the efficacy of the tsetse immune system [16]. N-acetyl glucosamine specific trypanocidal lectin is secreted during feeding, and trypanosomes need to successfully evade this lectin activity to establish in the midgut of the tsetse fly [17]. 

Because of their distinct reproductive biology, tsetse flies are recalcitrant to germ-line transformation [18]. *Sodalis glossinidius* is the only gamma proteobacterial tsetse endosymbiont to be cultured and is thus amenable to genetic modification [19]. A paratransgenic approach using *S. glossinidius* as a delivery system for trypanocidal components is currently of considerable interest to generate a trypanosome resistant tsetse fly [20]. Hence, investigation of the interactions between trypanosomes and *S. glossinidius* and, therefore, their influence on tsetse can provide new insights to design new vector control strategies.

In previous studies analysing natural tsetse fly populations, the relationship between *S. glossinidius* and trypanosomes varies with respect to tsetse fly species and trypanosome species/subspecies. For instance, in tsetse flies from Maasai Mara National Reserve, Kenya, there was a statistically significant relationship in *G. pallidipes* tsetse flies but not in *G. swynnertoni* [21]. There were no significant relationships found between *Sodalis* and trypanosomes in *G. brevipalpis, G. morsitans morsitans* and *G. pallidipes* tsetse flies from Luambe National Park, Zambia [22], but significant associations were found in *G. m. morsitans* from western Zambia [23]. *Sodalis glossinidius* has been shown to be positively associated with *T. congolense* and *T. b. rhodesiense* in *G. m. morsitans* [17]*, T. b. gambiense* and *T. b. brucei* in *G. p. gambiensis* [24] and *T. congolense* Forest, *T. brucei* s. l. and *T. b. gambiensis* in *G. p. palpalis* [25]. These results show that the relationship between *Sodalis* and the presence of trypanosomes varies depending on geographic areas, and there is lack of information about the tripartite relationship between *Sodalis*, trypanosomes and wild caught tsetse flies in Kafue National Park (KNP) in Zambia. As such, we conducted a tsetse fly survey to determine the prevalence and identify trypanosome species circulating in tsetse flies and to assess the associations between *Sodalis* and trypanosomes in this area. 

## 2. Materials and Methods

### 2.1. Study Area and Tsetse Fly Collection

**Figure 1:**
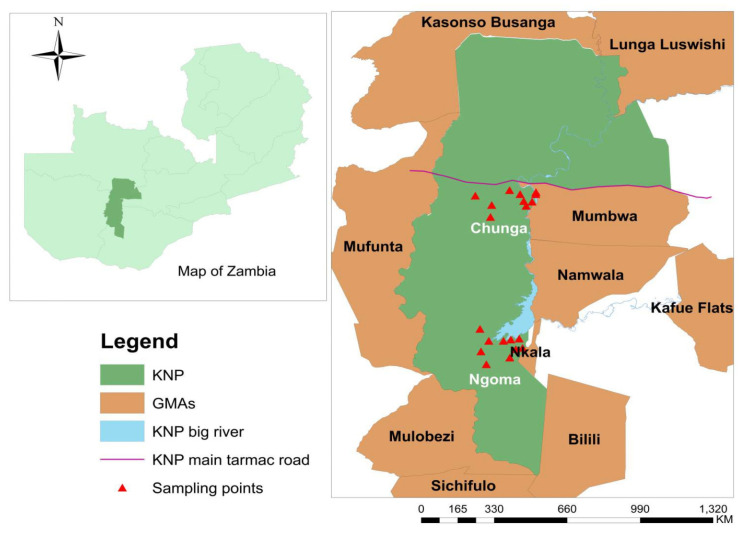
Map of the study area. Note: KNP = Kafue National Park, GMA = Game Management Area.

Tsetse fly samples were collected from the KNP ecosystem which is situated between 14°03″ S and 16°43″ S and 25°13″ E and 26°46″ E (Figure 1). The KNP ecosystem is the largest conservation area in Zambia and covers approximately 68,000 km^2^ of the country. It is the oldest and the largest national park (22,480 km^2^) in Zambia and is surrounded by 45,406 km^2^ of game management areas, which stretch over four provinces [26]. The park is rich in animal and natural diversity and forms one of the main important terrestrial ecosystems in Africa [27]. The study was carried out at the Chunga and Ngoma sampling locations within the KNP ecosystem and 150 km apart. The Chunga sampling site is located approximately 150 km from Mumbwa town and it is situated on the Kafue River. The sampling points in this area are covered by thicket vegetation. The Ngoma sampling site is located 26 km from Itezhi tezhi on the Kafue River and close to Itezhi tezhi Dam. The vegetation type at the Ngoma sampling points were thicket and miombo woodlands. 

The number of tsetse flies required for analysis of trypanosomes was estimated using previous findings of 26.85% prevalence in the same study area [28]. Based on this, the minimum number of tsetse flies was 301, and we caught 326 tsetse flies during the tsetse survey which were used for the analysis. 

Six Epsilon traps containing 3-n-propylphenol, octanol and 4-methylphenol at a ratio of 1:6:12 and an open 300 mL bottle containing acetone at the entrance to the trap were deployed under the tree sheds between September and December 2021. All the traps were deployed between 17:00 and 18:00, and the captured tsetse flies were collected by visiting traps at about 12 h intervals daily between 6:00 and 7:00 and 17:00 and 18:00. The geographic coordinates were recorded for each trap. ArcMap in ArcGIS was used to record the spatial locations of the sampling points on the map (Figure 1). The trapped tsetse flies were counted and grouped into teneral and non-teneral flies, as previously described elsewhere [29]. The teneral flies were discarded. The non-teneral flies were stored individually in a 1.5 mL Eppendorf tubes with silica beads and were transported to the University of Zambia, School of Veterinary Medicine Laboratory for DNA extraction and further analysis. 

### 2.2. Sex and Species Determination of Tsetse Flies

Morphological characterization was used to sort out the sex and species of the captured tsetse flies. The species and sex were identified using a stereomicroscope based on standard published keys [29]. 

### 2.3. DNA Extraction

Each tsetse fly was transferred to a new 2 mL microcentrifuge tube and smashed for 70 s at 3500 rpm in a Micro Smash^TM^ MS-100R bead cell disrupter (TOMY MEDICO. Ltd., Tokyo, JAPAN) using five 3-mm diameter zirconium beads. DNA was extracted from the homogenate of each tsetse fly using QIAGEN DNeasy Blood and Tissue Kit following the manufacturer’s instructions (Qiagen Sciences, Hilden, Germany). The DNA was quantified using a NanoDrop^TM^ 1000 Spectrophotometer (Thermo Scientific, Waltham, MA, USA), and DNA samples were stored at −80 °C until PCR analysis.

### 2.4. PCR for the Identification of African Trypanosomes and Sodalis glossinidius

All PCR reactions were conducted using OneTaq ^®^Quick-Load^®^ 2X Master Mix with Standard Buffer (20 mM Tris-HCI (pH 8.9 @ 25 °C), 1.8 mM MgCl_2_, 22 mM NH_4_Cl, 22 mM KCl, 0.2 mM dNTPs, 5% glycerol, 0.06% IGEPAL^®^ CA-630, 0.05% Tween^®^ 20, 25 units/mL OneTaq DNA Polymerase) (NEW ENGLAND BioLabs Inc., Ipswich, MA, USA). PCR reactions for both trypanosomes and *S. glossinidius* were carried out in a 10 μL reaction volume containing 5 μL of One Taq ^®^Quick-Load^®^ 2X Master Mix, 3.2 μL of nuclease free water, 0.4 μL from 10 μM of each forward and reverse primer and 1 μL of template DNA. Initial screening for the presence of trypanosome parasites was conducted using ITS1 CF and BR primers that target the internal transcribed spacer 1 (ITS1) (Table 1) [30]. The PCR conditions were an initial step at 94 °C for 5 min, followed by 35 cycles of 94 °C for 40 s, 58 °C for 40 s, 72 °C for 90 s, and final extension at 72 °C for 5 min. However, ITS1 primer has low sensitivity against *T. vivax* species [31]. To solve this problem, *T. vivax* specific primers (TVIV 1 and TVIV 2) were used with the same PCR conditions except the annealing temperature, which was 60 °C [32].

When the ITS1 PCR generated a PCR product of between 500 bp and 800 bp band sizes, *T. congolense* subgroup-specific PCR were conducted to differentiate the subgroup Kilifi, Forest and Savannah using subgroup-specific primers (Table 1) with PCR conditions of an initial step at 94 °C for 5 min, followed by 35 cycles of 94 °C for 30 s, 55 °C for 30 s, 72 °C for 90 s, and final extension step at 72 °C for 5 min. All tsetse flies which showed band sizes between 250 and 500 bp were subjected to PCR that differentiate pathogenic trypanosome species (*T. b. rhodesiense, T. b. brucei*, *T. simiae, T. simiae* Tsavo and *T. godfreyi*) using species-specific primers (Table 1). To check the human infective trypanosome species (*T. b. rhodesiense*), the serum resistance associated (SRA) gene PCR was performed using an amplification program with an initial denaturation step at 95 °C for 15 min followed by 35 cycles of 94 °C for 1 min, 68 °C for 1 min, 72 °C for 1 min and a final extension step of 72 °C for 10 min. 

The presence of *S. glossinidius* in all tsetse flies was determined using the primer pair GPO1F and GPO1R, which amplifies the 1200 bp product of the extrachromosomal plasmid, GPO1, of *Sodalis* [14]. The amplification program was initiated with an initial step at 94 °C for 5 min, followed by 35 amplification cycles with denaturation step of each cycle at 94 °C for 1 min, an annealing step at 55 °C for 1 min, and an extension step of 72 °C for 1 min followed by a final extension step at 72 °C for 10 min.

All PCR reactions included appropriate positive and negative controls. The PCR products were size-separated by electrophoresis in 1x TAE buffer (40 mM Tris, 20 mM acetic acid, 1 mM EDTA, pH 8.0) (BioConcept Ltd., Allschwil, Switzerland) on 1.5% agarose gel (CSL-AG100, Cleaver Scientific Ltd., Rugby, UK), stained with Ethidium bromide and visualized under UV light. Amplicon sizes were determined relative to a 100 bp DNA ladder.

### 2.5. Data Analysis

Data were entered into MS-Excel ^®^ and analysed using R software version 4.1.0 [37]. The prevalence of *S. glossinidius* and trypanosomes were estimated using frequencies. The Chi-square test or Fisher’s Exact test where appropriate were used to compare the prevalence of trypanosomes and *S. glossinidius* with sex, species and collection site of tsetse flies. They were also used to assess whether the presence of *S. glossinidius* was associated with trypanosome infections. All the statistics were considered significant at *p* ≤ 0.050.

## 3. Results

### 3.1. Tsetse Fly Survey 

**Figure 2:**
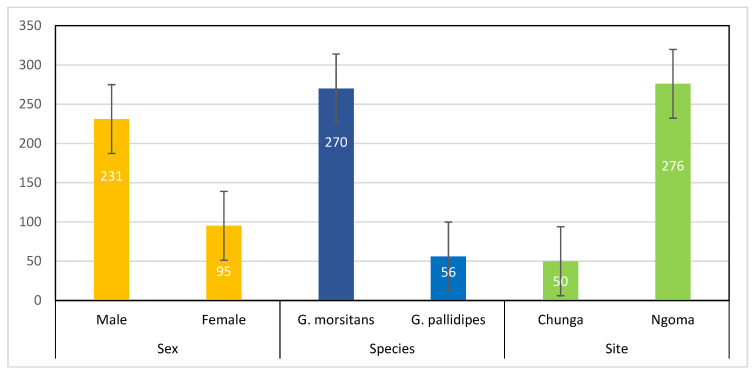
Tsetse fly population structure according to sex, species and sampling site. Note: Error bars correspond to 95% confidence interval.

A total of 326 tsetse flies were trapped: 231 (70.9%, 95% CI: 65.7–75.5%) were male and 95 (29.1%, 95% CI: 24.5–34.3%) were female tsetse flies. Out of the total tsetse samples collected, 270 (82.8%, 95% CI: 78.35–86.53) were *G. morsitans* and 56 (17.2%, 95% CI: 13.47–21.65) were *G. pallidipes*, and 50 (15.3%, 95% CI: 11.83–19.85) were from Chunga and 276 (84.7%, 95% CI: 80.35–88.17) were from Ngoma sampling locations (Figure 2).

### 3.2. Prevalence of Trypanosomes in Tsetse Flies

Of 326 tsetse fly samples subjected to PCR using general ITS1 primers, 63 (19.3%) were found with DNA of at least one trypanosome species, indicating an overall prevalence of 19.3% (95% CI: 15.41–23.96). The differences in prevalence of trypanosomes between *G. morsitans* and *G. pallidipes*, Chunga and Ngoma, and male and female tsetse flies are shown in Table 2. The prevalence of trypanosomes was significantly higher in Ngoma than in Chunga (χ ^2^ = 6.73, *p* = 0.009) (Table 2). There were no statistically significant differences in the prevalence of trypanosomes between male and female (χ^2^ = 0.53, *p* = 0.467) and between *G. morsitans* and *G. pallidipes* (χ^2^ = 0.19, *p* = 0.661) tsetse flies (Table 2).

Six trypanosome species were detected in all tsetse flies. These were *T. vivax, T. simiae, T. congolense, T. godfreyi, T. simiae* Tsavo, and *T. brucei brucei*. *Trypanosoma vivax* (6.4%, 95% CI = 4.25–9.65) was the most prevalent, and *T. b. brucei* (2.5%, 1.25–4.77) was the least. Table 3 and Table 4 summarise the prevalence of each trypanosome species that was identified with respect to species, sex and sampling site of tsetse flies. There were no significant differences in the prevalence of trypanosome species detected between species, sex and sampling sites (Table 3 and Table 4).

Among 13 tsetse flies which were positive for *T. congolense,* eight had the *T. congolense* Kilifi subgroup, two had the *T. congolense* Forest subgroup, and one had *T. congolense* Savannah. Two tsetse flies had mixed *T. congolense* subgroups. Of these, one had a mixed infection of *T. congolense* Kilifi and *T. congolense* Forest. The other had *T. congolense* Forest mixed with *T. congolense* Savannah. 

No human infective *T. b. rhodesiense* was detected in either species of tsetse flies.

**Figure 3:**
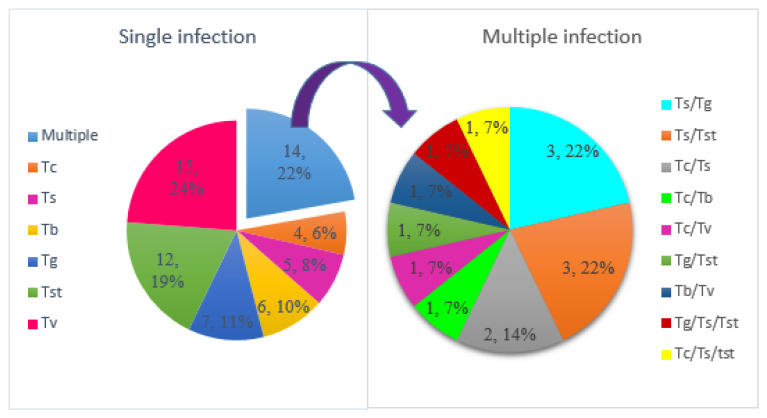
Distribution of single and multiple infections of trypanosome in tsetse flies. Note: Tc_*T. congolense*, Tb_*T. b. brucei*, Tg_*T. godfreyi,* Ts*_T. simiae,* Tst*_T. simiae* Tsavo, and Tv*_T. vivax*.

Most tsetse flies were infected with a single trypanosome species (49/63, 77.8%), followed by tsetse flies infected by two trypanosome species (12/63, 19.0%) and tsetse flies that had three trypanosome species (2/63, 3.2%) (Figure 3). Multiple infections with two trypanosome species included three tsetse flies that had a mixture of *T. simiae* and *T. godfreyi*, three tsetse flies that had *T. simiae* and *T. simiae* Tsavo, two tsetse flies infected with *T. congolense* and *T. simiae*, one tsetse fly with *T. congolense* and *T. vivax*, one tsetse fly contained *T. congolense* mixed with *T. b. brucei*, one tsetse fly with *T. b. brucei* and *T. vivax* and one tsetse fly with *T. simiae* Tsavo and *T. godfreyi*. Triple infections were found in two tsetse flies which had *T. simiae*/*T. godfreyi*/*T. simiae* Tsavo and *T. simiae*/*T. congolense*/*T. simiae* Tsavo.

### 3.3. Prevalence of S. glossinidius in Tsetse Flies

The overall prevalence of *S. glossinidius* from the 326 tsetse flies was estimated to be 21.8% (95% CI: 17.64–26.57). The prevalence was higher in female (24.2%; 95% CI: 16.71–33.72) than in male tsetse flies (20.8%; 95% CI: 16.05–26.47), although this was not statistically significant (χ^2^ = 0.46, *p* = 0.670). It was also slightly higher in *G. morsitans* (22.2%; 95% CI: 17.67–27.55) than in *G. pallidipes* (19.6%; 95% CI: 11.34–31.84) but not statistically significantly different (χ^2^ = 0. 0.18, *p* = 0.495). The prevalence between the two sampling sites was not significantly different (χ^2^ = 0.62, *p* = 0.432), although it was slightly higher in Chunga (26.0%: 95% CI: 15.87–39.55) than in Ngoma (21.0%; 95% CI: 16.62–26.20).

The prevalence of *S. glossinidius* in each tsetse species was examined based on sampling location and the sex of tsetse flies (Table 5). In *G. morsitans*, there were no statistically significant differences in the prevalence of *S. glossinidius* between Chunga and Ngoma (χ^2^ = 0.97*, p* = 0. 324) or between male and female flies (χ^2^ = 0.01*, p* = 0.943) (Table 5). There were also no significant differences in the prevalence of *S. glossinidius* between Chunga and Ngoma (*p* = 1.000) or between male and female (*p* = 0.142) *G. pallidipes* flies (Table 5).

### 3.4. Association between S. glossinidius and Presence of African Trypanosomes

Out of 63 trypanosome-infected flies, 47.6% of the flies were also co-infected with *S. glossinidius,* while the remaining flies were devoid of *S. glossinidius*. The analysis performed on the overall dataset indicated that there was a significant association between tsetse flies harboring *S. glossinidius* and tsetse flies infected with trypanosomes (χ^2^ = 30.61, *p* < 0.001). The association varied between sampling sites, with tsetse flies from Ngoma showing a statistically significant association (χ^2^ = 30.39, *p* < 0.001), whereas tsetse flies from Chunga showed no statistically significant association (*p* = 0.162). In *G. morsitans*, twenty-five out of sixty tsetse flies with *S. glossinidius* were infected with trypanosomes, and there was a statistically significant association (χ^2^ = 26.12, *p* < 0.001) between the two pathogens. From the eleven *S. glossinidius* positive *G. pallidipes* tsetse flies, five had trypanosome co-infections, and the association was statistically significant (*p* = 0.045). A statistically significant association was also observed between *S. glossinidius* and trypanosome prevalence in male tsetse flies (χ^2^ = 28.42, *p* < 0.001), but no such association was observed in female tsetse flies (*p* = 0.058) (Table 6).

Among the tsetse flies infected with *T. simiae* Tsavo, *T. simiae*, and *T. vivax*, the co-infection rates with *S. glossinidius* were 60.0%, 46.7%, and 47.6%, respectively. Among the tsetse flies infected by *T. congolense*, co-infection with *S. glossinidius* was 30.8%, and for *T. b. brucei* and *T. godfreyi*, the co-infection rate was 50.0% for both (Table 7). There was a statistically significant association between *S. glossinidius* and *T. vivax* (*p* = 0.006), *T. simiae* (*p* = 0.025), *T. simiae* Tsavo (*p* = 0.009), and *T. godfreyi* (*p* = 0.027), but no such association was detected between *S. glossinidius* and *T. congolense* (*p* = 0.491) and *T. b. brucei* (*p* = 0.072) (Table 7).

## 4. Discussion

### 4.1. Prevalence of Trypanosome Species

The aim of this study was to determine the prevalence of trypanosome and *S. glossinidius* in tsetse flies collected from the Chunga and Ngoma areas of the Kafue National Park. The study also intended to determine whether an association between trypanosomes and *S. glossinidius* existed in these tsetse flies. Both *Sodalis* and trypanosomes were prevalent in tsetse flies obtained from the study area. The prevalence of trypanosomes found in this study was similar to the 17.4% prevalence reported from Ghana [38] but lower than previously reported results from national park and wildlife reserve areas such as Nkhotakota Wildlife Reserve and Liwonde Wildlife Reserve, Malawi [39,40], Luambe National Park, Luangwa Valley, and the Kafue ecosystem, Zambia [22,28,41], Nech Sar National Park, Ethiopia [42], Shimba Hills National Reserve, Kenya [43], and Santchou Wildlife Reserve, Cameroon [44]. However, the prevalence obtained from the current study was higher compared with the 0.8% reported from Ghana [28], 2.40% from Kenya [21], 3.4% from Tanzania [45], 6.31% from Zimbabwe [46], 10.7% from Uganda [47], and 11.4% from Kenyan coastal forests and South Africa [48]. These differences could be explained by differences in geographic location, the availability of potential tsetse species and presence of appropriate vertebrate hosts. 

In the current work, six trypanosome species were detected with *T. vivax* being the most prevalent. This is in agreement with another study that reported a high prevalence of *T. vivax* in tsetse flies and cattle in the same study area [28]. The result is also in agreement with other studies in Zambia [23] and other parts of Africa [21,45]. Other trypanosome species detected were *T. simiae*, *T. congolense, T. godfreyi*, *T. simiae* Tsavo, and *T. b. brucei*. The higher prevalence of *T. vivax* in tsetse flies compared with other trypanosome species may be due to the differences in development cycles as *T. vivax* completes its entire development only in proboscis, whereas *T. congolense*, *T. simiae, T. godfreyi*, and *T. simiae* Tsavo in complete it in the proboscis and midgut and *T. b. brucei* in the midgut and salivary gland, which can be affected by low pH, protease activity and lectins [49,50]. 

In this study, there were no significant differences in the levels of trypanosome infections between male and female tsetse flies. This result is similar to a finding from Chad [51] and other experimental studies [52] but in disagreement with previous studies from Nigeria [53] and Côte d’Ivoire [54] where researchers reported higher prevalences of trypanosomes in females than male tsetse flies. It is also in contrast to other experimental studies in the same tsetse species [55] in which males had higher trypanosome infectivity than their female counterparts.

Despite a recent report of a HAT case in an adult male [56] and the presence of *T. b. rhodesiense* in vervet monkey, sable antelope, buffalo [57] and in cattle [28] from the KNP ecosystem, no human infective trypanosome species were detected in this study. Although *T. b. rhodesiense* was not detected in the current study, the presence of the most competent tsetse fly vectors of *T. b. rhodesiense* (*G. morsitans* and *G. pallidipes*) in KNP and the high prevalence of *T. b. rhodesiense* previously reported in wildlife [57] and cattle [28] in the area indicate an existing risk of emergence of HAT, so coordinated surveillance and control efforts are required in the study area.

### 4.2. Prevalence of S. glossinidius 

The overall prevalence of *S. glossinidius* estimated from this study was lower than the 31.3% prevalence reported from southwest Nigeria [58] and the 34.0% prevalence reported from the Shimba Hills and Nguruman regions in Kenya [43]. However, the prevalence in the current study was higher than that in the Maasai Mara National Reserve (6.6%), a wildlife–human–livestock interface in Kenya [21] and that reported in the Shimba Hills National Reserve (15.9%), a wildlife–human–livestock interface on Kenya’s south coast [48]. 

The difference in the prevalence of *S. glossinidius* in relation to species of tsetse flies was not statistically significant. In *G. morsitans*, the prevalence of *S. glossinidius* was higher than the 17.5% reported in Luambe National Park, in eastern Zambia [22] and the 15.9% obtained from Western Zambia [23]. However, this value is lower than the prevalence of 29.6% that was reported from Zimbabwe [59] and the 28.6% reported from Adamawa region of Cameroon [60]. In this study, the prevalence of *S. glossinidius* in *G. pallidipes* was higher than the 1.4%, 6.5%, 15.9%, and 16% recorded in Luambe National Park, Zambia [22], Maasai Mara National Reserve, Kenya [21], Shimba Hills National Reserve, Kenya [48], and tsetse flies collected from Zimbabwe [59], respectively. This prevalence was, however, lower than the 83.3% in *G. pallidipes* collected from Tanzania [59]. These differences may be linked to environmental and ecological variations between sampling areas which can highly affect the biology of tsetse flies and the presence of different *S. glossinidius* genotypes and trypanosomes [61].

In this study, no significant difference in the prevalence of *S. glossinidius* between male and female tsetse flies was detected. This finding is in agreement with other studies by Dennis et al. [22] and Mathew [59] which reported similar results. Data analysed for individual tsetse species also indicated no significant difference in the prevalence of *S. glossinidius* between sexes of *G. morsitans* and *G. pallidipes*. 

### 4.3. Association between S. glossinidius and Trypanosome Infections in Tsetse Flies

From the overall data analysed, the co-infection rate between *S. glossinidius* and trypanosomes in this study were lower than the 37% rate reported from the “Faro and Déo” division of the Adamawa region of Cameroon [60] and the rate of 32.2% in two historical HAT foci in Cameroon [25], but higher than 2% co-infection rate in Kenyan coastal forests [48]. This result indicates that the presence of *S. glossinidius* is not absolutely necessary for tsetse flies to be infected by trypanosomes, but the presence of *S. glossinidius* would highly favor such infections. 

In the current study, significant associations were found between the presence of *S. glossinidius* and the presence of trypanosomes in tsetse flies. This maybe an indication that presence of *S. glossinidius* favors trypanosome infections in tsetse flies. This is in agreement with other studies from Cameroon [25], western Zambia [23] and Kenya [21], where significant associations were reported between *S. glossinidius* and trypanosome infections in different tsetse fly species. However, there was variation in the association of *Sodalis* and trypanosomes between tsetse fly species, sex and sampling locations. 

There were no large differences in the proportions of co-infected tsetse flies with *Sodalis* and trypanosomes in *G. morsitans* and *G. pallidipes* (9.3% and 8.9%, respectively). Significant associations were found between *Sodalis* and trypanosome infections in *G. morsitans* and *G. pallidipes* tsetse fly species. These findings are in line with other studies conducted in *G. m. centralis* [23], *G. pallidipes* [48], *G. pallidipes* and *G. swynnertoni* [21] and *G. p. palpalis* [25] where significant associations between *S. glossinidius* and trypanosome infections were reported in the respective tsetse fly species. These findings support the hypothesis that presence of *S. glossinidius* increases the susceptibility to and establishment of trypanosome infections in *G. morsitans* and *G. pallidipes* tsetse flies. However, this is in contrast to the findings of a study of *G. morsitans* and *G. pallidipes* in tsetse flies from Luambe National Park, Zambia [22] where no association was found. In addition to the presence or absence of *Sodalis*, this difference may be due to a difference in *S. glossinidius* genotype which may affect the association between *S. glossinidius* and trypanosome infections, as described by Geiger et al. [62]. Based on the sex of tsetse flies, there was a significant association between *S. glossinidius* and trypanosome infections in males, but no significant association was observed between the endosymbiont and trypanosome infections in females. These differences may be due to the small number of female tsetse flies collected for the endosymbiont and trypanosome infections. Separate analyses of the data for each sampling site indicate there are differences in the statistical association between the endosymbiont and trypanosome infections, where a statistically significant association was observed for the Ngoma sampling site, but not for the Chunga sampling site. This difference could be due to the low trypanosome infection rate and small number of tsetse flies captured at the Chunga sampling site. 

The associations between *S. glossinidius* and each trypanosome species infection were also examined. The result of this analysis clearly indicates that significant associations were found between *S. glossinidius* and *T. simiae*, *T. vivax*, *T. simiae* Tsavo, and *T. godfreyi*. However, there were no significant associations between *S. glossinidius* and *T. congolense* and *T. b. brucei*. This difference is probably due to *S. glossinidius* affecting the establishment of trypanosomes depending on the trypanosome genotype [24]. 

One limitation of this study was that we could not discriminate between established infections and residual bloodmeal contamination as PCR detects trypanosome DNA in the fly bloodmeal, which can remain in the tsetse tissues after the death of the parasite. This could lead to higher prevalence estimates of trypanosomes than the true prevalence and further affect the association between *S. glossinidius* and trypanosomes. 

## 5. Conclusions

Investigation of *S. glossinidius* confirmed the presence of the endosymbiont in *G. morsitans* and *G. pallidipes* tsetse flies. The study confirmed the circulation of pathogenic trypanosome species in *G. morsitans* and *G. pallidipes* in the study area. The results also show that some tsetse flies were infected by both the endosymbiont and trypanosome, whereas others were infected by either the endosymbiont or trypanosome only, or had no infection at all. The association between *S. glossinidius* and trypanosome infections is complex and seems to vary according to tsetse fly sex and trypanosome species, with *T. simiae*, *T. simiae* Tsavo, *T. vivax,* and *T. godfreyi* being significantly associated with *S. glossinidius*. To increase understanding about the tripartite association and to use *S. glossinidius* as a potential target for genetic transformation to control vectors of trypanosomes, further research on genetic comparisons between *S. glossinidius* detected in tsetse flies co-infected with trypanosomes and *S. glossinidius* detected without trypanosome infections is required.

## Figures and Tables

**Table 1:** Primers used.

Organism	Target Gene	Primer Name	Primer Sequence (5’ to 3’)	Amplicon Size (bp)	Annealing Temperature (°C)	Reference
*Trypanosoma* spp.	ITS1 rDNA	ITS1 CF	CCGGAAGTTCACCGATATTG	Variable	58	[30]
		ITS1 BR	TTGCTGCGTTCTTCAACGAA			
*T. congolense* Kilifi	Satellite DNA monomer	TCK 1	GTGCCCAAATTTGAAGTGAT	294	55	[33]
		TCK 2	ACTCAAAATCGTGCACCTCG			
*T. congolense* Forest	Satellite DNA monomer	TCF 1	GGACACGCCAGAAGGTACTT	350	55	[33]
		TCF 2	GTTCTCGCACCAAATCCAAC			
*T. congolense* Savannah	Satellite DNA monomer	TCS 1	CGAGAACGGGCACTTTGCGA	316	55	[33]
		TCS 2	GGACAAAGAAATCCCGCACA			
*T.b. rhodesiense*	SRA gene	SRA284 F	ATAGTGACAAGATGCGTACCAACGC	284	68	[34]
		SRA284 R	AATGTGTTCGAGTACTTCGGTCACCT			
*T. vivax*		TVIV-F	CTGAGTGCTCCATGTCCCAC	142	60	[32]
		TVIV-R	CCACCAGAACACCAACCTGA			
*T. brucei s. l.*		TBR 1	GAATATTAAACAATGCGCAG	164	58	[33]
		TBR 2	CCATTTATTAGCTTTGTTGC			
*T. simiae*		TSM1	CCGGTCAAAAACGCATT	437	58	[33]
		TSM2	AGTCGCCCGGAGTCGAT			
*T. simiae* Tsavo		TST1	GTCCTGCCACCGAGTATGC	450	58	[35]
		TST2	CGAGCATGCAGGATGGCCG			
*T. godfreyi*		DGG1	CTGAGGCTGAACAGCGACTC	149	58	[36]
		DGG2	GGCGTATTGGCATAGCGTAC			
*S. glossinidius*	GPO1	GPO1F	TGAGAGGTTCGTCAATGA	1200	55	[14]
		GPO1R	ACGCTGCGTGACCATTC			

**Table 2:** Prevalence of African trypanosomes in relation to sex, species and sampling site of tsetse flies.

Variable	Categories	*n*	Prevalence (%)	95% CI	Statistical Analysis
Sampling site	Chunga	50	6.0	2.06–16.22	χ^2^ = 6.73, *p* = 0.009 *
	Ngoma	276	21.7	17.28–26.98	
Sex	Male	231	20.4	15.66–26.00	χ^2^ = 0.53, *p* = 0.467
	Female	95	16.8	10.64–25.62	
Species	*G. morsitans*	270	18.9	14.67–23.98	χ^2^ = 0.19, *p* = 0.661
	*G. pallidipes*	56	21.4	12.71–33.82	
*G. morsitans*	Male	190	20.0	14.93–26.26	χ^2^ = 0.30, *p* = 0.583
	Female	80	16.3	9.75–25.84	
*G. pallidipes*	Male	41	22.0	12.00–36.71	*p* = 1.000 ^a^
	Female	15	20.0	7.05–45.19	
Chunga	*G. morsitans*	47	6.4	2.19–17.16	*p* = 1.000 ^a^
	*G. pallidipes*	3	0.0	0.00–56.15	
Ngoma	*G. morsitans*	223	21.5	16.64–27.38	χ^2^ = 0.03, *p* = 0.859
	*G. pallidipes*	53	22.6	13.45–35.53	

*n*: sample size; 95% CI: confidence interval; ^a^ Fisher’s exact, * statistically significant.

**Table 3:** Prevalence of trypanosomes species identified according to tsetse fly species.

Trypanosome Species	Overall (*n* = 326)	* G. morsitans * (*n* = 270)	* G. pallidipes * (*n* = 56)
* T. congolense *	4.0% (2.35–6.70)	4.4% (2.56–7.61)	1.8% (0.32–9.45)
		* p * = 0.705 ^a^	
* T. vivax *	6.4% (4.25–9.65)	6.3% (3.97–9.85)	7.1% (2.81–16.98)
		* p * = 0.768 ^a^	
* T. b. brucei *	2.5% (1.25–4.77)	1.9% (0.79–4.26)	5.4% (1.84–14.61)
		* p * = 0.142 ^a^	
* T. simiae *	4.6% (2.81–7.45)	4.8% (2.84–8.06)	3.6% (0.98–12.12)
		* p * = 1.000 ^a^	
* T. godfreyi *	3.7% (2.12–6.32)	3.3% (1.76–6.21)	5.4% (1.84–14.61)
		* p * = 0.440 ^a^	
* T. s. * Tsavo	3.1% (1.67–5.55)	3.7% (2.02–6.68)	0.0% (0.0–6.42)
		* p * = 0.221 ^a^	

*n =* number of tsetse flies; ^a^ Fisher’s exact.

**Table 4:** Prevalence of trypanosome species according sex and sampling site of tsetse flies.

Trypanosome Species	Sex		Sampling Site	
	Male (*n* = 231)	Female (*n* = 95)	Chunga (*n* = 50)	Ngoma (*n* = 276)
*T. congolense*	4.8% (2.68–8.32)	2.1% (0.58–7.35)	2.0% (0.35–10.50)	4.4% (2.50–7.44)
	*p* = 0.360 ^a^		*p* = 0.700 ^a^	
*T. vivax*	6.5% (3.97–10.44)	6.3% (2.93–13.10)	2.0% (0.35–10.50)	7.3% (4.74–10.93)
	*X*^2^ = 0.004, *p* = 0.953		*p* = 0.220 ^a^	
*T. b. brucei*	2.6% (1.20–5.55)	2.1% (0.58–7.35)	2.0% (0.35–10.50)	2.5% (1.23–5.14)
	*p* = 1.000 ^a^		*p* = 1.000 ^a^	
*T. simiae*	4.3% (2.37–7.78)	5.3% (2.27–11.73)	2.0% (0.35–10.50)	5.1% (3.05–8.33)
	*p* = 0.773 ^a^		*p* = 0.483 ^a^	
*T. godfreyi*	3.9% (2.06–7.24)	3.2% (1.08–8.88)	2.0% (0.35–10.50)	4.0% (2.24–6.99)
	*p* = 1.000 ^a^		*p* = 0.700 ^a^	
*T. s.* Tsavo	3.0% (1.48–6.12)	3.2% (1.08–8.88)	0.0% (0.0–7.13)	3.6% (1.98–6.54)
	*p* = 1.000 ^a^		*p* = 0.371 ^a^	

*n* = number of tsetse flies; ^a^ Fisher’s exact.

**Table 5:** Prevalence of *S. glossinidius* in *G. morsitans* and *G. pallidipes* based on sex and sampling site.

Species	Location	*n*			Prevalence (95% CI)			
		M	F	Total	M	F	Overall	*p*-Value
*G. morsitans*	Chunga	22	25	47	27.3% (13.15–48.15)	28.0% (14.28–47.58)	27.7% (16.94–41.76)	χ^2^ = 0.97, *p* = 0.324
	Ngoma	168	55	223	21.4% (15.90–28.24)	20.0% (11.55–32.36)	21.1% (16.24–26.90)	
	Total	190	80	270	25.3% (19.62–31.89)	22.5% (14.73–32.79)	22.2% (17.67–27.55)	
					χ^2^ = 0.01, *p* = 0.943			
*G. pallidipes*	Chunga	2	1	3	0.0	0.0	0.0	*p* = 1.000 ^a^
	Ngoma	39	14	53	15.4% (7.25–29.73)	35.7% (16.34–61.24)	20.8% (12.00–33.46)	
	Total	41	15	56	14.6% (6.88–28.44)	33.3% (15.18–58.29)	19.6% (11.34–31.84)	
					*p* = 0.142 ^a^			

*n* = number of tsetse flies checked, M = Male, F = Female, *p* = *p*-value, ^a^ Fisher’s exact.

**Table 6:** Association between *S. glossinidius* and the presence of trypanosomes.

	Overall		*G. morsitans*		*G. pallidipes*		Male		Female		Chunga		Ngoma	
	T+	T−	T+	T−	T+	T−	T+	T−	T+	T−	T+	T−	T+	T−
S+	30	41	25	35	5	6	23	26	7	16	2	11	28	30
S−	33	222	26	184	7	38	24	159	9	63	1	36	32	190
	χ^2^ = 30.61, *p* < 0.001		χ^2^ = 26.12, *p* < 0.001		*p* = 0.045 ^a^		χ^2^ = 28.42, *p* < 0.001		*p* = 0.058 ^a^		*p* = 0.162 ^a^		χ^2^ = 30.39, *p* < 0.001	

T+ = Trypanosome positive, T− = Trypanosome negative, S+ = *Sodalis* positive, S− = *Sodalis* negative, *p* = *p*-value, ^a^ Fisher’s exact.

**Table 7:** Association between *S. glossinidius* and *Trypanosoma* species detected in tsetse flies.

	*T. congolense*		*T. vivax*		*T. b. brucei*		*T. simiae*		*T. simiae* Tsavo		*T. godfreyi*	
	Tc+	Tc−	Tv+	Tv−	Tbb+	Tbb−	Ts+	Ts−	Tst+	Tst−	Tg+	Tg−
S+	4	67	10	61	4	67	7	64	6	65	6	66
S−	9	247	11	244	4	251	8	247	4	251	6	249
	*p* = 0.491 ^a^		*p* = 0.006 ^a^		*p* = 0.072 ^a^		*p* = 0.025 ^a^		*p* = 0.009 ^a^		*p* = 0.027 ^a^	

S+ = *Sodalis* positive, S− = *Sodalis* negative, Tc+ = *T. congolense* positive, Tc− = *T. congolense* negative, Tv+ = *T. vivax* positive, Tv− = *T. vivax* negative, Tbb+ = *T. b. brucei* positive, Tbb− = *T. b. brucei* negative, Ts+ = *T. simiae* positive, Ts− = *T. simiae* negative, Tst+ = *T. simiae* Tsavo positive, Tst− = *T. simiae* Tsavo negative, Tg+ = *T. godfreyi* positive, Tg− = *T. godfreyi* negative; ^a^ Fisher’s exact, *p* = *p*-value.

## Data Availability

All the datasets used and/or analysed in this study are available from the corresponding author on reasonable request.
